# Absence of vaccine-enhanced RSV disease and changes in pulmonary dendritic cells with adenovirus-based RSV vaccine

**DOI:** 10.1186/1743-422X-8-375

**Published:** 2011-07-29

**Authors:** Anja Krause, Yaqin Xu, Sara Ross, Wendy Wu, Ju Joh, Stefan Worgall

**Affiliations:** 1 Department of Genetic Medicine, Weill Medical College of Cornell University, New York, New York, USA; 2 Department of Pediatrics, Weill Medical College of Cornell University, New York, New York, USA

## Abstract

The development of a vaccine against respiratory syncytial virus (RSV) has been hampered by the risk for vaccine-enhanced RSV pulmonary disease induced by immunization with formalin-inactivated RSV (FIRSV). This study focuses on the evaluation of vaccine-enhanced pulmonary disease following immunization with AdF.RGD, an integrin-targeted adenovirus vector that expresses the RSV F protein and includes an RGD (Arg-Gly-Asp) motif. Immunization of BALB/c mice with AdF.RGD, resulted in anti-RSV protective immunity and induced increased RSV-specific IFN-γ T cell responses compared to FIRSV. RSV infection 5 wk after immunization with FIRSV induced pulmonary inflammatory responses in the lung, that was not observed with AdF.RGD. Additionally, In the FIRSV-immunized mice following infection with RSV, pulmonary DC increased and Tregs decreased. This suggests that distinct responses of pulmonary DC and Tregs are a features of vaccine-enhanced RSV disease and that immunization with an RGD-modified Ad vaccine does not trigger vaccine-enhanced disease.

## Introduction

RSV is a leading cause of severe viral respiratory disease in infants and children [1]. A major obstacle in the development of an RSV vaccine has been vaccine-enhanced disease triggered by immunization with inactivated virus antigen [2,3]. This aberrant immune-mediated response is characterized by infiltration of neutrophils and eosinophils, increased complement-fixing antibody titers and lymphoproliferative responses [4-7]. The exact mechanism has not been fully elucidated. An altered pattern of CD4 lymphocyte activation with eosinophil recruitment and Th2-type-predominant cytokine production (IL-4, IL-5, IL-13 and eotaxin) suggests an aberrant immune-mediated response skewed towards Th2 responses [4,8-10]. This is supported by data demonstrating that the disease following RSV infection can be transferred by RSV G protein-specific CD4 T cells and also occurs in the absence of CD8 T cells or IFN-γ [11-13]. RSV-specific CD8 T cells can even inhibit the disease [7,13,14]. Initially, the RSV G protein itself was implicated based on studies using vaccinia virus expressing the membrane-anchored part or the entire G protein for immunization [15-17]. Recently, it has been suggested that the pathway of antigen processing, rather than the antigenic content, is responsible [4,7]. In addition, low avidity anti-RSV antibodies, which may have resulted from poor activation of toll-like receptors, have been observed in mice following immunization with FIRSV [18], the most commonly used model to study vaccine-enhanced RSV disease [4,19-21].

The RSV F protein, one of the main capsid proteins that confers protective immunity against RSV, has been a major target in RSV vaccine development [22]. Vaccination with the F protein generates helper T cell responses that are Th1 in character [23]. Adenovirus (Ad)-based gene delivery systems are promising platforms for genetic vaccines due to their ability to act as immune system adjuvants and to induce strong cellular and humoral responses against the virus and the transgene [24,25] and have been used as experimental vaccines against RSV [26-32]. In part, the effectiveness of Ad-based vaccines results from the ability of Ad vectors to transfer genes to antigen presenting cells *in vivo*, particularly dendritic cells (DC) [33-37]. The immune response can be further enhanced to a more Th1-dominant response by modification of Ad capsid proteins to infect DC more efficiently [38-40]. The primary interaction of Ad with cells *in vitro *is through the knob domain of fiber to the coxsackievirus and adenovirus receptor (CAR) on the target cell [41,42]. A secondary interaction occurs between the RGD (Asp-Arg-Gly) motif in the penton base and integrins. The addition of an RGD motif (in addition to that found in the penton base) enhances infection of DC, which express low levels of CAR and high levels of surface integrins [38-40].

Virus-specific humoral and cellular adaptive immune responses are responsible for protection and recovery from RSV infection. Lung DC, as part of the pulmonary innate immune system, recognize the infection, evoke anti-viral responses and modulate the Th1/Th2 balance [43-47]. There are two major subsets of DC in the mouse, (1) the CD11b^+^, CD11c^high ^conventional DC (cDC), and (2) the CD11b^low-^, CD11c^high^, B220^+ ^plasmacytoid DC (pDC) [48-52]. Resting lung cDC have been implicated in initiating a pro-allergic Th2 response in the lung and in requiring special cytokine signals to induce Th1 response, whereas pDC seem to have a primary role in producing IFN-α in response to viral infections and in promoting a Th1 response by blocking the pulmonary immune environment against a Th2 response [43,50,53-56]. PDC balance the T helper cell responses through cross-talk with regulatory T cells (Tregs). The number of pDC and cDC is increased in lung and draining lymph nodes following experimental RSV infection in mice [55,57-59] as well as in nasal washings of RSV-infected infants [60]. RSV-stimulated cDC seem to have immunostimulatory effects on both Th1 and Th2 responses [57]. RSV-stimulated pDC have direct antiviral activity through the release of IFN-α (2). Depletion of pulmonary pDC leads to an exaggerated Th2 response to RSV [55,56], whereas increasing the number of pDC by recombinant Flt3 ligand leads to an increased Th1 response [50]. Increasing cDC while depleting pDC leads to enhanced Th2-type pathology [50]. We hypothesized that the lung DC subsets stimulated in response to RSV infection in immunized animals would depend upon the T-helper cell type induced by immunization.

The present study demonstrates that immunization with the capsid-modified Ad vector AdF.RGD compared to immunization with FIRSV, at doses inducing comparable levels of anti-RSV neutralizing and protective immunity, leads to a more pronounced RSV-specific Th1 response and does not prime for vaccine-induced enhanced RSV disease. Furthermore, immunization with AdF.RGD or FIRSV results in a distinct response of pulmonary DC and Tregs that may be useful for the characterization of vaccine-enhanced pulmonary RSV disease.

## Results

### Immunization with AdF.RGD Leads to Enhanced Th1-Type anti-RSV Immunity and Improved Protection against RSV

**Figure 1:**
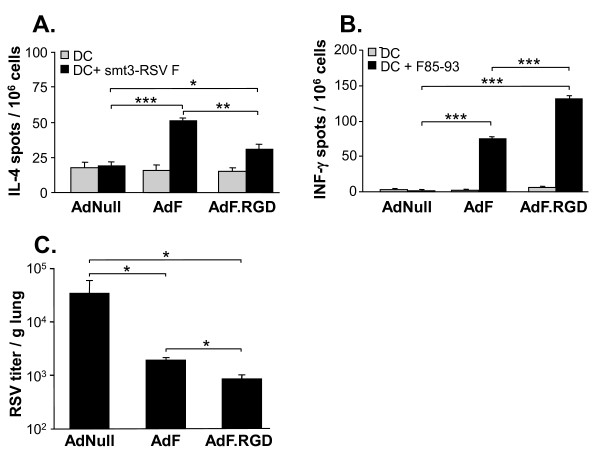
**Frequency of RSV F protein-specific Th2-type CD4 and CD8 T cells and immunity against RSV in BALB/c mice following intramuscular immunization with AdF or AdF.RGD (10**^**10 **^**pu/mouse)**. **A, B**. Ten days following immunization, spleen CD4 and CD8 T cells were co-cultured with splenic DC alone or with DC pulsed with recombinant smt3-RSV F protein or F peptide (DC-smt3-RSV F; DC-F85-93, respectively) and RSV F-specific cytokine responses were measured after 48 h by ELISPOT assay. **A**. RSV-specific IL-4 production in CD4 T cells; **B**. F epitope-specific production in CD8 T cells. **C**. RSV titer in the lungs of mice challenged by intranasal administration of RSV (10^6 ^cfu) 4 wk after immunization. Data represent mean ± SD of 5 animals/group from one of two independent experiments. ***, ** and * denote significance of p < 0.001, p < 0.01 and p < 0.05, respectively.

We first evaluated if immunization with AdF.RGD could further polarize the anti-F immune response towards Th1 and improve the protection against RSV in comparison to immunization with the capsid-unmodified vector AdF. BALB/c mice were immunized intramuscularly with either AdF, AdF.RGD or AdNull and IL-4 responses in CD4 T cells and IFN-γ responses in CD8 T cells against the recombinant RSV F protein (smt3-RSV F) and the H-2^d^-restricted F epitope (F85-93, KYKNAVTEL), respectively, were evaluated in lymphocytes isolated from spleen 10 days following immunization (Figure 1A,B). F protein-specific IL-4 in CD4 T cells were induced both by AdF and AdF.RGD (p < 0.001 and p < 0.03 compared to AdNull, respectively; Figure 1A). However, the AdRGD-induced response was lower compared to AdF-induced response (p < 0.002). In contrast, the RSV-F epitope-specific IFN-γ response in CD8 T cells was increased in the mice immunized with AdF.RGD compared to mice immunized with AdF (p < 0.001; Figure 1B). This suggested that the RGD modification of the AdF vector increased the anti-RSV Th1 response and decreased the Th2 response. To evaluate if the immunization induces sufficient immunity to provide protection against a subsequent pulmonary challenge with RSV, BALB/c mice were immunized with AdF, AdF.RGD, or AdNull and challenged 5 wk later with RSV intranasally. Immunization with both AdF and AdF.RGD resulted in reduced RSV titers in the lung compared to the AdNull group (p < 0.05, both comparisons; Figure 1C). Interestingly, immunization with AdF.RGD resulted in further reduction in the RSV lung titers than immunization with AdF (p < 0.01). This suggests that the RGD modification results in increased protective immunity against RSV. Based on these results, the AdF.RGD vector was used for the following experiments.

### Immunization with AdF.RGD Leads to Improved Humoral anti-RSV Immunity Without Inducing Vaccine-enhanced Disease

**Figure 2:**
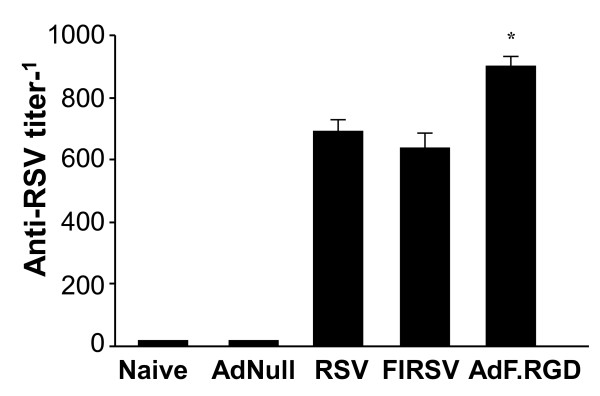
**Protective immunity induced by AdF.RGD**. BALB/c mice were immunized intramuscularly with AdNull, AdF.RGD (both at 10^10 ^pu), formalin-inactivated RSV (FIRSV, 10^5 ^pfu) or intranasally with RSV (10^6 ^pfu) and neutralizing serum anti-RSV Titer was measured 4 wk following immunization. Data represent mean ± SD of 5 animals/group from one of two independent experiments.* denotes significance p < 0.05.

Humoral immune responses were evaluated following immunization with AdF.RGD in direct comparison to immunization with RSV and FIRSV, a vaccine that triggers vaccine-enhanced RSV disease. BALB/c mice immunized with AdF.RGD, RSV or FIRSV all had increased neutralizing anti-RSV titers in the serum after 4 wk compared to mice that had received the control AdNull vector (p < 0.001; Figure 2). AdF.RGD induced neutralizing anti-RSV antibodies at higher levels compared to live RSV and FIRSV-immunized mice (p < 0.05). There was no difference in neutralizing anti-RSV titer between RSV and FIRSV-immunized animals (p > 0.1).

**Figure 3:**
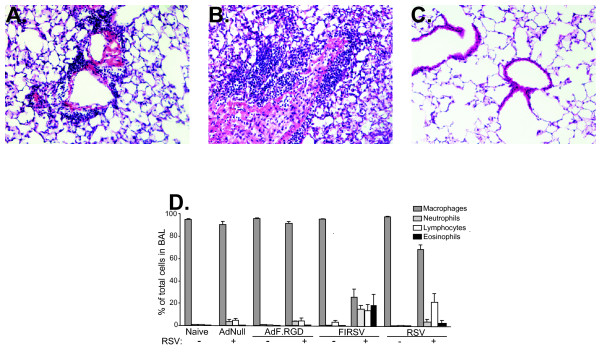
**Absence of RSV-induced inflammatory responses in lung and BAL following immunization with AdF.RGD**. BALB/c mice were immunized subcutaneously with AdNull, AdF.RGD (both at 10^10 ^pu), formalin-inactivated RSV (FIRSV, 10^5 ^pfu) or intranasally with RSV (10^6 ^pfu). Five wk later the mice were challenged intranasally with RSV (10^6 ^pfu) and lungs were harvested after 6 days. **A-C**. Lung histology (H+E stain): **A**. RSV, **B**. FIRSV, **C**. AdF.RGD. **D**. Quantification of cells in BAL. Data for **D **are shown as mean ± SEM of duplicate measurements of n = 4 mice/group and represent one of two independent experiments.

**Figure 4:**
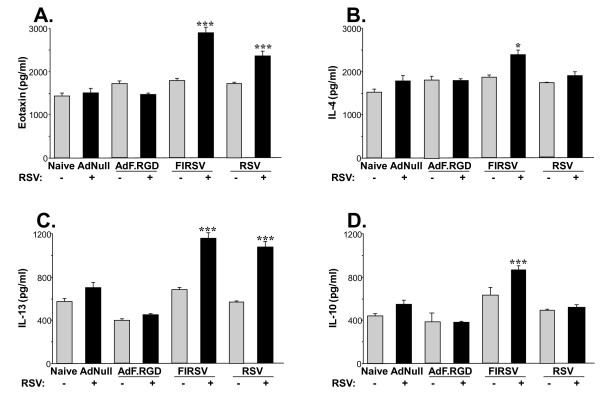
**Absence of RSV-induced inflammatory responses in lung and BAL following immunization with AdF.RGD**. BALB/c mice were immunized subcutaneously with AdNull, AdF.RGD (both at 10^10 ^pu), formalin-inactivated RSV (FIRSV, 10^5 ^pfu) or intranasally with RSV (10^6 ^pfu). Five wk later the mice were challenged intranasally with RSV (10^6 ^pfu) and lungs were harvested after 6 days. **A-D**. Cytokine levels in homogenates: **A**. Eotaxine levels in lung homogenate. **B**. IL-4 levels in lung homogenate. **C**. IL-13 levels in lung homogenate. **D**. IL-10 levels in lung homogenate Data are shown as mean ± SEM of duplicate measurements of n = 4 mice/group and represent one of two independent experiments.*** and * denote significance of p < 0.001 and p < 0.05, respectively.

To evaluate if immunization with AdF.RGD induces vaccine-enhanced RSV lung disease, mice were immunized with AdF.RGD, AdNull, RSV or FIRSV and then challenged with RSV at 5 wk post-immunization. Vaccine-enhanced RSV disease was assessed 6 days following RSV infection by: (1) lung histology and (2) the cellular composition of the bronchioalveolar lavage (BAL), (Figure 3), and (3) the Th2-type cytokines eotaxin, IL-4, IL10 and IL-13 in lung homogenate (Figure 4). Pronounced inflammation, consistent with vaccine-enhanced disease, was seen in the lungs of animals that had been immunized with FIRSV and had been challenged with RSV (Figure 3B). Vaccination with RSV induced moderate inflammation in the lung after RSV challenge with less infiltration of inflammatory cells compared to the FIRSV immunized animals after RSV challenge (Figure 3A). In contrast, only minimal inflammatory changes were observed in the mice immunized with AdF.RGD (Figure 3C). Analysis of the cell differential in the BAL showed an increase in eosinophils, lymphocytes and neutrophils FIRSV-immunized animals and an increase in lymphocytes in the RSV-immunized animals that had been challenged with RSV compared to those that had received AdF.RGD or AdNull (Figure 3D). Only a mild increase in the number of lymphocytes and neutrophils was observed in AdF.RGD and AdNull-immunized animals challenged with RSV compared to unchallenged animals (Figure 3D). Consistent to these findings, the levels of inflammatory cytokines like IL-4, IL-10, Il-13 and eotaxin in lung homogenate were increased in mice that had been immunized with FIRSV prior to RSV challenge compared to Naive, AdNull and AdF.RGD lung homogenates (eotaxin: p < 0.001, Figure 4A; IL-4: p < 0.05, Figure 4B; IL-13 and IL-10: p < 0.001, Figure 4C and 4D, respectively). Immunization with RSV prior to RSV challenge induced similar elevated eotaxin and IL-13 levels in the lung as seen in the FIRSV-immunized animals (Figure 4A and 4C, respectively), whereas IL-4 and IL-10 levels were not increased compared to naive mice or mice that had received AdNull or AdF.RGD. This suggests that immunization with AdF.RGD does not trigger these features of vaccine-induced lung disease.

### Immunization with AdF.RGD Leads to Cellular and Protective anti-RSV Immunity

**Figure 5:**
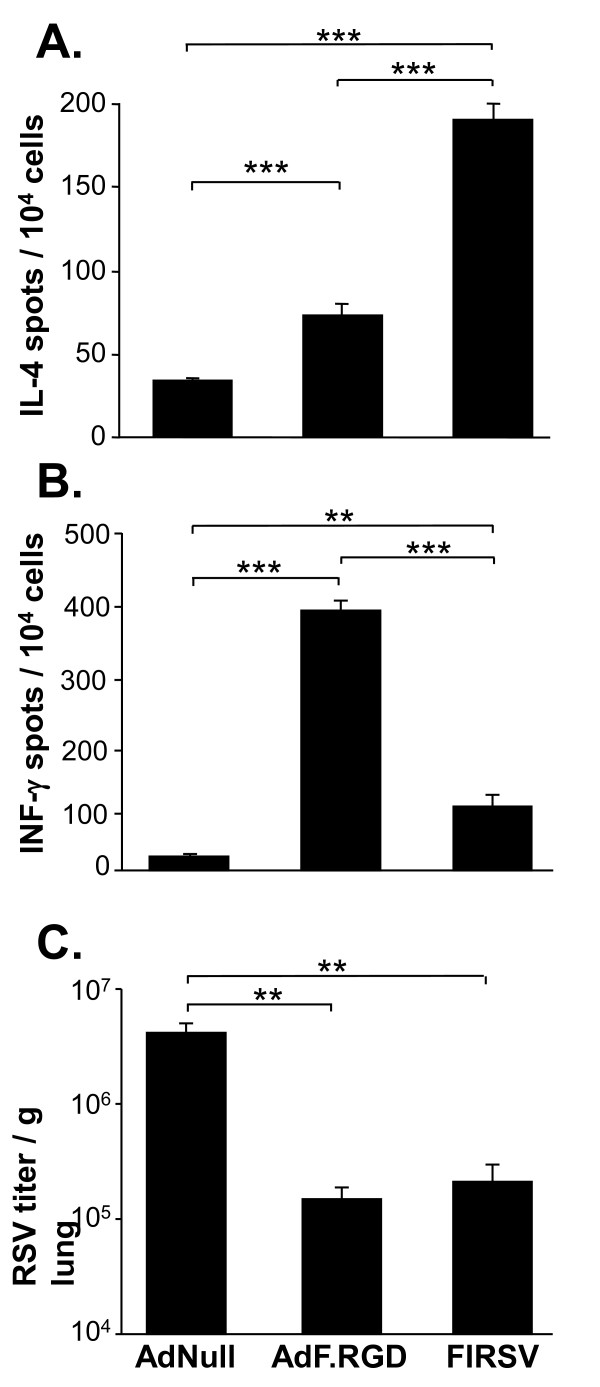
**Frequency of RSV-specific Th2-type CD4 and CD8 T cells and protection**. BALB/c mice were immunized subcutaneously with AdNull (10^10 ^pu/mouse), AdF.RGD (10^10 ^pu/mouse) or FIRSV (10^5 ^pfu) **A, B**. Ten days following immunization spleen CD4 and CD8 T cells were cultured with splenic DC pulsed with UV-inactivated RSV (10^5 ^pu/ml) for 48 h. RSV-specific cytokine response were determined by ELISPOT assay. **A**. RSV-specific IL-4 production in CD4 T cells. **B**. RSV-specific IFN-γ production in CD8 T cells. **C**. RSV titer in the lungs of mice challenged by intranasal administration of RSV (10^6 ^cfu) 4 wk after immunization. Data represent mean ± SD of 5 animals/group from one of two independent experiments. *** and ** denote significance of p < 0.001 and p < 0.01, respectively.

Cellular and protective immune responses were evaluated following immunization with AdF.RGD in direct comparison to immunization with FIRSV, the vaccine that resulted in vaccine-enhanced RSV disease. BALB/c mice were immunized with AdF.RGD, AdNull or FIRSV and the systemic anti-RSV cellular immune response was evaluated 10 days after vector administration (Figure 5). The number of RSV-specific CD4 T cells secreting IL-4 from the spleen cells of mice immunized with AdF.RGD was reduced compared to mice immunized with FIRSV (p < 0.001; Figure 5A). In contrast, AdF.RDG induced a higher RSV-specific IFN-γ response in CD8 T cells than FIRSV (p < 0.001; Figure 5B). Protection against RSV challenge 4 wk after immunization was similar following immunization with AdF.RGD and FIRSV (p > 0.5) compared to the AdNull control group (p < 0.002, both comparisons; Figure 5C). These data indicate that, at the doses used, immunization with AdF.RGD and FIRSV leads to comparable levels of protective immunity, but that mice immunized with AdF.RGD have a higher Th1 and lower Th2 response than FIRSV-immunized mice and subsequently do not develop vaccine-enhanced lung disease.

### Characterization of Lung DC following Infection with RSV in AdF.RGD-Immunized Mice

**Figure 6:**
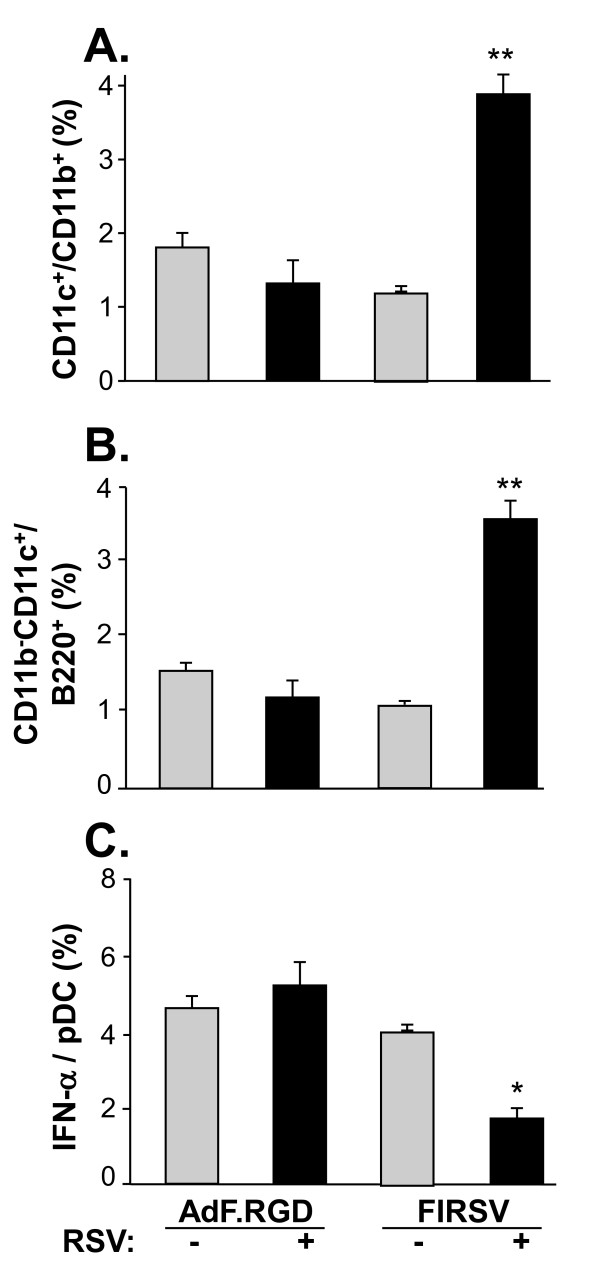
**Lung DC and IFN-α secretion following infection with RSV in immunized mice**. BALB/c mice were immunized subcutaneously with AdF.RGD (10^10 ^pu/mouse) or FIRSV (10^5 ^pfu). **A-C**. Five wk later the mice were challenged intranasally with RSV (10^6 ^pfu) for 6 days and the lung suspensions were analyzed for cDC (CD11b^+^,CD11c^high^), pDC (CD11b^low^, CD11c^high^, B220^+^, PDCA-1^+^) and IFN-α secretion by flow cytometry and intracellular cytokine staining. **A**. cDC. **B**. pDC. **C**. IFN-α secretion by pDC. Data are presented as mean ± SEM of 4 mice/group from one of two independent experiments.** and * denote significance of p < 0.01 and p < 0.05, respectively.

RSV infection results in changes in lung DC [50,56-61]. The lung cDC and pDC subsets regulate Th1 and Th2 responses, with pDC balancing the Th1/Th2 response via activation of Treg cells and consequently shifting the cDC-promoted Th2 response towards Th1 response [62]. To further analyze the DC composition in the lung following RSV infection, lung suspensions were prepared 6 days after intranasal administration of RSV and cDC and pDC populations were analyzed by flow cytometry. Consistent with prior reports [55,57-59], the number of CD11c/CD11b-positive cDC and the percentage of PDCA-1-positive pDC (CD11b^-^/CD11c^+^) increased after RSV infection (data not shown). To analyze the mechanism by which lung DC respond to RSV infection in AdF.RGD-immunized mice compared to mice that received the formalin-inactivated RSV vaccine, lung DC subsets were isolated 6 days following RSV infection from mice that had been immunized with AdF.RGD or FIRSV 5 wk prior to RSV infection. The cDC population did not significantly change between AdF.RGD and AdF.RGD plus RSV animals (p > 0.09; Figure 6A), indicating that vaccination with AdF.RGD does not lead to a change in the cDC population after RSV challenge. In contrast, cDC increased following RSV infection in the mice immunized with FIRSV compared to all the other groups (p < 0.01, all comparisons; Figure 6A). A similar pattern was observed in the pDC population with the highest increase in the FIRSV-immunized mice challenged with RSV (p < 0.01; Figure 6B). Since viral infections are strongly controlled by IFN-α secretion by pDC [62], we analyzed the percentage of IFN-α-positive pDC in the lungs of vaccinated mice following challenge with RSV. IFN-α positive pDC in the lungs of mice that had been immunized with AdF.RGD, AdF.RGD plus RSV and FIRSV were similar (p > 0.1, all comparisons; Figure 6C). However, there was a significant decrease in the IFN-α secretion in pDC population in the mice immunized with FIRSV and challenged with RSV (p < 0.02; Figure 6C). This indicates that pDC and cDC are increased in vaccine-induced RSV disease following immunization with FIRSV and that their imbalance could induce a Th2-promoting environment.

**Figure 7:**
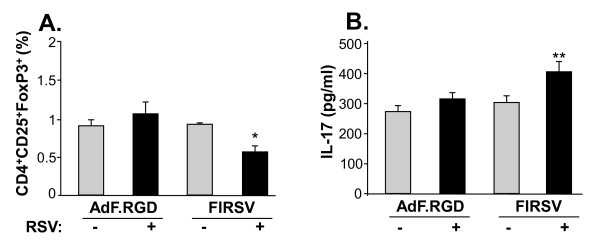
**Treg cells (CD4**^**+**^**, CD25**^**bright**^**, FoxP3**^**+**^**) and IL-17 level following infection with RSV in immunized mice**. BALB/c mice were immunized subcutaneously with AdF.RGD (10^10 ^pu/mouse) or FIRSV (10^5 ^pfu). **A, B**. Five wk later the mice were challenged intranasally with RSV (10^6 ^pfu) for 6 days and lung suspensions were analyzed for Treg cells by flow cytometry and IL-17 cytokine level. **A**. Treg cells. **B**. IL-17. Data are presented as mean ± SEM of 4 mice/group from one of two independent experiments.* and ** denote significance of p < 0.05 and p < 0.01, respectively.

Since pDC and cDC function as activators and regulators for Tregs (CD4^+^, CD25 ^bright^, FoxP3^+^), a T cell population that controls the outcome of Th1 and Th2 responses [62,63], we analyzed if the CD4^+^CD25^+^FoxP3^+ ^is affected during FIRSV-induced vaccine disease and if this effect is abolished following immunization with AdF.RGD. The Treg population did not differ in the mice that had been immunized with AdF.RGD and mice that had been immunized with AdF.RGD and subsequently been challenged with RSV (p > 0.08; Figure 7A). In contrast, animals that had been immunized with FIRSV and were challenged with RSV showed a significant decrease in the Treg population (p < 0.05, all comparisons; Figure 7A). As there is a reciprocal relationship between Treg cells and IL-17 expression by Th17 cells, we evaluated the IL-17 cytokine level in the lung (Figure 7B). No significant differences were observed between mice that had been immunized with AdF.RGD and mice that had been immunized with AdF.RGD and challenged with RSV (p > 0.09; Figure 7B). In contrast, animals that had been immunized with FIRSV and were challenged with RSV showed a significant increase in the IL-17 level in the lung (p < 0.01, all comparisons; Figure 7B). Overall, this data suggests that the lung DC and Treg populations in vaccinated mice show distinct changes following RSV challenge, dependent upon whether the vaccine induces enhanced disease.

## Discussion

Based on the exaggerated inflammatory Th2 response primed by the formalin-inactivated RSV vaccine more than 40 years ago, it has been thought that an efficient and safe vaccine against RSV should induce a Th1-dominant response. Our present study shows that, compared to FIRSV, an integrin-targeted Ad vector expressing the RSV F protein induces higher levels of serum neutralizing anti-RSV antibodies with equivalent protection against RSV, but showed a decreased Th2 response and did not induce RSV disease. Immunization with FIRSV followed by RSV infection induces an imbalance in the pulmonary cDC and pDC population, diminished anti-viral activity in pDC, and a lower frequency of regulatory T cells that was not present following immunization with AdF.RGD.

### Modification of Ad Vector to Increase Th1 Responses

The effectiveness of Ad-based vaccines is thought to be based on the efficient infection of a variety of cells *in vivo*, subsequently the expression of a pathogen-specific protein encoded by the Ad expression cassette in these cells, as well as Ad vectors functioning as adjuvants [24,25]. Ad vectors have the capability to infect DC and this is critical for the induction of a strong anti-Ad and anti-transgene immune response [33-37]. Generating protective immunity against viruses like RSV requires the generation of humoral and cellular immunity by the vaccine. One attractive feature of Ad for genetic vaccination is the capability to modify the Ad capsid to enhance immune responses [38-40]. Targeting Ad to antigen-presenting cells by adding the RGD integrin-binding motif to the fiber knob enhances anti-transgene cellular immune responses in mice and predominantly skews cellular immune responses towards Th1, a prerequisite to develop successful anti-viral vaccines [38-40]. AdF.RGD induced neutralizing anti-RSV titer, a strong anti-RSV Th1-dominant cellular response and protected from RSV after challenge.

### Vaccine-induced RSV Disease

Although many vaccine approaches, including live attenuated, viral and bacterial vectored and adjuvanted subunit vaccines, have been evaluated in rodent and primate models, there is currently no approved RSV vaccine. Screening for a live attenuated RSV vaccine candidate with the right balance of attenuation and immunogenicity has been hampered by a lack of suitable *in vitro *and *in vivo *models capable of accurately predicting attenuation in naive infants [64]. Vaccine-induced RSV disease following immunization with formalin-inactivated preparations of RSV, based on the initial clinical FIRSV vaccine, has been extensively studied in animal models responses [4-7,19,21]. Studies in cotton rats, vaccinated with FIRSV and then challenged with RSV showed infiltration with neutrophils, macrophages and lymphocytes and increased Th2-type cytokines like IL-4, IL-10, IL-13 and eotaxin in the lung, indicating a Th2 bias in enhanced inflammation [5,65]. The central role of T cells in the augmented lung pathology has been elucidated using the BALB/c mouse model. It has been shown that CD4 T cells are crucial to the immunopathogenesis of vaccine-augmented RSV disease [4,66,67], and that RSV-specific antibodies (in the absence of CD4 and CD8 T cells) are not sufficient to cause disease enhancement [4,66]. It has recently been reported that immunization with FIRSV induces low avidity anti-RSV antibodies due to poor activation of toll-like receptors that could consequently result in an aberrant Th2 immune response [18]. Therefore, skewing to a Th1-type pattern of cytokine production by priming with live RSV prevented subsequent enhanced disease [67]. Studies using recombinant vaccinia viruses expressing various RSV proteins have pointed to the G protein as a potential crucial factor in the induction of vaccine-enhanced disease [68]. Since the challenge lies in inducing a strong and protective T cell response, while avoiding the pathological consequences of unbalanced Th1/Th2 responses, Ad vectors, which tend to induce strong Th1 responses, are a promising tool for a RSV vaccine. The addition of the RGD motif to the fiber further increased the Th1 response against the F protein and did not induce vaccine-enhance diseased.

### Role of DC in Vaccine-induced RSV Disease

It has been reported that the number of cDC and pDC is increased during RSV infection and even after apparent resolution [55,57-61]. Collectively, these data support the hypothesis that recruitment of recently attracted DCs in the lung leads to maintenance and control of the immune response in the lung, even after the actual infection and inflammation in the lung is resolved [43,50,54-56].

To date, there has been no description of the role of lung DC subsets in respiratory viral infections during the onset of vaccine-induced RSV disease. RSV-infected mice developed airway hyperreactivity as measured by a strong increase in airway resistance in response to methacholine. Depletion of pDCs around the time of RSV infection enhanced peribronchial and perivascular inflammatory infiltrates consisting of mononuclear cells and increased pro-inflammatory cytokines and decreased anti-viral secretion [55,56]. Mice that were immunized with FIRSV and subsequently challenged with RSV showed similar patterns of lung DC subset imbalance, resulting in down-regulation of anti-viral secretion in pDC as well as decreased numbers of Treg cells. Although the subset populations were increased after RSV challenge, decreased Treg cells and elevated levels of IL-17 levels indicated a Th2-type milieu which may reflect an imbalance of pDC and consequently cDC function and which was not seen following immunization with AdF.RGD. The analysis of lung DCs and Tregs may thus be useful in the evaluation for vaccine-enhanced disease of new RSV vaccines.

## Methods

### Adenovirus Vectors

The recombinant Ad vectors used in this study are E1a-, partial E1b- and partial E3- vectors based on the Ad5 genome. AdF contains the human cytomegalovirus (CMV) intermediate early promoter/enhancer, the sequenced F protein cDNA of the RSV A2 strain, same that was used for further challenge experiments, and the simian virus 40 (SV40) polyA stop signal as an expression cassette inserted into the E1 region. The F protein cDNA was kindly provided by P. Collins (Bethesda, MD). AdF.RGD has the high-affinity RGD sequence GCDCRGDCFCA incorporated at the COOH-terminal end of the fiber protein [42]. The AdNull control vector contains no transgene in the expression cassette. The vectors were used on the basis of equal number of physical particle concentration and were propagated and purified as described previously [69].

### Mice

Female BALB/c mice were obtained from Taconic Farms (Tarrytown, NY). The animals were housed under specific pathogen-free conditions and used at 6 to 8 wk of age. The mice were immunized by intramuscular injection of AdF.RGD, AdNull (both at 10^10 ^particle units; pu), FIRSV (10^5 ^pfu), or by intranasal administration of RSV (10^5 ^pfu). All animal studies were conducted under protocols reviewed and approved by the Weill Cornell Institutional Animal Care and Use Committee.

### RSV

The RSV strain used for immunization and protection experiments was A2 (VR-1540; ATCC). FIRSV was prepared from supernatant of RSV-infected HEp-2 cells, combined with paraformaldehyde (1:1600 dilution of 37-38% stock, Sigma) to a final dilution of 1:4000, and stirred for 3 days at 37°C. The virus was then pelleted by ultracentrifugation, resuspended, and alum-precipitated [3,9,67].

### Cellular Immune Response

To assess the RSV F-specific cellular immune response following immunization, BALB/c mice were immunized by intramuscular administration of either AdF, AdF.RGD, AdNull (all at 10^10 ^pu), or FIRSV (10^5 ^pfu). Unimmunized mice served as additional controls. The frequency of antigen-specific T lymphocytes was determined 10 days following immunization in an IFN-γ- and IL-4-specific ELISPOT assay. MAIPS-45 plates (Millipore, Bedford, MA) were coated overnight at 4°C with 5 μg/ml of cytokine-specific capture antibodies [AN18 (IFN-γ) or 11B11 (IL-4); Mabtech, Stockholm, Sweden]. Spleen single cell suspensions served as the source for DC, CD4 and CD8 T cells. CD4 or CD8 T cells were purified by negative depletion using SpinSep T cell subset purification kits (StemCell Technologies, Vancouver, BC, Canada). Purity for CD4 and CD8 T cells was between 95-98%. Splenic DC were purified from naive animals to serve as antigen presenting cells by positive selection using CD11c MACS beads (Miltenyi Biotec, Auburn, CA) and double-purification over two consecutive MACS LS+ columns (Milentyi Biotec). The resulting DC purity was between 90-96%. DC (5 × 10^6^/ml) were either pulsed for 3 hr with the H-2^d ^restricted F epitope (F85-93, KYKNAVTEL, 100 μM, purity confirmed by high-performance liquid chromatography), incubated with purified recombinant RSV F protein (100 μg/ml), or UV-inactivated RSV (10^5 ^pu/ml) in complete RPMI medium supplemented with 10 mM Hepes, pH 7.5 (BioSource International, Camarillo, CA) and 10^-5 ^M b-mercaptoethanol (Sigma-Aldrich). The recombinant RSV F protein was produced from a bacterial expression vector. A bacterial expression vector (pSmt3-RSV F) was constructed, by cloning the PCR amplified RSV F gene (forward primer: 5'-CCC CGA TCC ACA ATG GAG TTG CTA ATC CTC-3'; reverse primer: 5'-A TAA CGT AAA TCA TTG ATT TTC GAA CCC-3') into the petSUMO expression vector (Invitrogen) and the recombinant fusion protein was purified by Ni-chelating affinity chromatography according to the manufacturer's protocol (Prebound, Qiagen, Valencia, CA). Prior to addition of responder T cells, antibody-coated plates were blocked with complete RPMI medium supplemented with 10 mM HEPES, pH 7.4 (BioSource International) and 10^-5 ^M β-mercaptoethanol (Sigma-Aldrich) for 3 h. CD4 (2 × 10^5^) or CD8 (10^5^) T cells were incubated with splenic DC, pulsed with either recombinant RSV F protein, F85-93 peptide, RSV, or no antigen at a T cell: DC ratio of 6:1 for 48 h. Following washing, biotinylated anti-IFN-γ or anti-IL-4 detection antibodies (both at 1 μg/ml, Mabtech) were added and the plates were incubated for 2 h at 37°C, followed by a streptavidin-alkaline phosphatase conjugate (Vectastain-ABC peroxidase kit, Vector Laboratories, Burlingame, CA) and a 3-amino-9-ethylcarbazole substrate (Sigma) for spot detection. The spots were counted by computer-assisted ELISPOT image analysis (Zellnet Consulting, New York, NY).

### Protection of Mice from Intranasal Challenge with RSV

To evaluate the protection against RSV infection following immunization, BALB/c mice were immunized by intramuscular administration of AdF.RGD, AdNull (both at 10^10 ^pu) or FIRSV (10^5 ^pfu), and were then challenged with RSV (10^6 ^pfu) by intranasal inoculation after 4 wk. Four days later the mice were sacrificed, the lungs were homogenized in 1 ml MEM and the homogenates centrifuged at 1200 rpm at 4°C for 10 min. Ten-fold serial dilutions of the lung homogenate supernatant were incubated in infection medium (MEM supplemented with 1% penicillin/streptomycin) on HEp2 cells for 3 h at 37°C. The medium was then replaced with 1% methylcellulose in MEM containing 5% fetal bovine serum and 1% penicillin/streptomycin). After 4 days, the cells were fixed with 4% paraformaldehyde, stained with 1% crystal violet and the plaques were counted under a microscope. The neutralization titer was calculated from the average plaques of four wells as the reciprocal of the highest dilution of serum that completely prevented RSV activity (>90%).

### Neutralizing RSV Titer

To evaluate serum anti-RSV neutralizing antibody titers, BALB/c mice were immunized by intramuscular administration of AdF.RGD, AdNull (both at 10^10 ^pu), FIRSV (10^5 ^pfu), or by intranasal administration of RSV (10^5 ^pfu). Sera was collected 4 wk post-administration and serial dilutions of the sera in infection medium were incubated with RSV (strain A2) for 1 h at 37°C. They were then incubated on HEp2 cells for 90 min at 37°C. The medium was then changed to 1% methylcellulose medium and incubated for 4 days. The cells were fixed and the titers quantified as outlined above.

### Vaccine-enhanced RSV Disease

To evaluate vaccine-enhanced RSV pulmonary disease, mice were immunized by intramuscular administration of AdF.RGD, AdNull (both at 10^10 ^pu), FIRSV (10^5 ^pfu), or by intranasal administration of RSV (10^5 ^pfu). The mice were challenged with RSV at 5 wk post-immunization and sacrificed 6 days later. Lungs were fixed in 4% paraformaldehyde at a constant pressure of 25 cm H_2_O for 4 hr. Histological sections were stained with hematoxylin and eosin and evaluated in a blinded study for inflammatory changes by light microscopy. A second experimental group of mice was sacrificed at 4 days following RSV infection for evaluation of cell content and IL-4, IL-10, IL-13, IL-17 and eotaxin concentrations by ELISA (R&D System, MN).

### Lung DC and Tregs

BALB/c mice were immunized with AdF.RGD, AdNull (all at 10^10 ^pu), FIRSV (10^5 ^pfu) or RSV (10^5 ^pfu) 5 wk prior to intranasal challenge with RSV (10^5 ^pu). Lungs were harvested 6 days post-challenge, digested for 20 min at 37°C with DNAseI and Collagenase (Sigma-Aldrich, MO), and passed through a cell strainer. Myeloid and lymphoid DC were characterized as CD11b^+^, CD11c^bright ^cells (conventional DC, cDC), and pDC were characterized as CD11b^low^, CD11c^bright^, B220^+ ^and confirmed as PDCA-1^+ ^cells by flow cytometry (BD Bioscience, CA). IFN-α secretion by pDC was determined by intracellular cytokine staining. Briefly, cells were fixed and permeabilized with Cytofix/Cytoperm reagent (BD Biosciences) for 20 min at 4°C, then washed twice in Perm/Wash solution (BD Biosciences). The cells were then stained (30 min, 4°C) for intracellular cytokine using phycoerythrin (PE)-conjugated monoclonal antibody against murine cytokine IFN-α. CD4^+^, CD25^bright^, FoxP3^+ ^Tregs from the lung were analyzed 6 days post-challenge with RSV by co-staining the lung suspensions with allophyocyanine-conjugated anti-murine CD4, phycoerythrin-conjugated anti-murine CD25 and fluorescein-conjugated anti-murine FoxP3 antibodies (BD Bioscience). Cells were then analyzed by flow cytometry using a FACSCalibur flow cytometer (Becton Dickinson, NJ).

### Statistics

The data are presented as mean ± standard error of the mean. Statistical analyses were performed using a non-paired two-tailed Student's t-test. Statistical significance was determined at p < 0.05.

## Competing interests

The authors declare that they have no competing interests.

## Authors' contributions

AK designed this study, participated in the experiments and drafted and edited the manuscript, YX carried out the experiments and participated in discussions, SR participated in the experiments, WW carried out the experiments, JJ participated in the experiments, SW designed this study and edited the manuscript. All authors read and approved the final manuscript.

## Note

^‡ ^These studies were supported, in part, by R01 A1059228.
